# C-Reactive Protein Levels in the Brugada Syndrome

**DOI:** 10.4061/2011/341521

**Published:** 2011-11-29

**Authors:** Aimé Bonny, Joelci Tonet, Manlio F. Márquez, Antonio De Sisti, Abdou Temfemo, Caroline Himbert, Fatima Gueffaf, Fabrice Larrazet, Ivo Ditah, Robert Frank, Françoise Hidden-Lucet, Guy Fontaine

**Affiliations:** ^1^Service de Cardiologie, Hôpital Saint Camille, 2 Rue des Pères Camilliens, 94366 Bry sur Marne, France; ^2^Unité de Rythmologie, Hôpital Pitié Salpêtrière, 47-83 Boulevard de l'Hôpital, 75651 Paris, France; ^3^Departamento de Electrofisiología, Instituto Nacional de Cardiología Ignacio Chávez, Juan Badiano 1, Col. Sección XVI, 14080 Mexico City, DF, Mexico; ^4^Faculté des Sciences du Sport, Allée P Grousset, 80025 Amiens, France; ^5^Service de Cardiologie, Hôpital d' Instruction des Armées Begin, 69 Avenue de Paris, 94300 Vincennes, France; ^6^Department of Internal Medicine, School of Medicine, Wayne State University, 5475 Woodward Avenue Detroit, MI 48202, USA

## Abstract

*Background*. Inflammation in the Brugada syndrome (BrS) and its clinical implication have been little studied. *Aims*. To assess the level of inflammation in BrS patients. *Methods*. All studied BrS patients underwent blood samples drawn for C-reactive protein (CRP) levels at admission, prior to any invasive intervention. Patients with a previous ICD placement were controlled to exclude those with a recent (<14 days) shock. We divided subjects into symptomatic (syncope or aborted sudden death) and asymptomatic groups. In a multivariable analysis, we adjusted for significant variables (age, CRP ≥ 2 mg/L). *Results*. Fifty-four subjects were studied (mean age 45 ± 13 years, 49 (91%) male). Twenty (37%) were symptomatic. Baseline characteristics were similar in both groups. Mean CRP level was 1,4 ± 0,9 mg/L in asymptomatic and 2,4 ± 1,4 mg/L in symptomatic groups (*P* = .003). In the multivariate model, CRP concentrations ≥ 2 mg/L remained an independent marker for being symptomatic (*P* = .018; 95% CI: 1.3 to 19.3). *Conclusion*. Inflammation seems to be more active in symptomatic BrS. C-reactive protein concentrations ≥ 2 mg/L might be associated with the previous symptoms in BrS. The value of inflammation as a risk factor of arrhythmic events in BrS needs to be studied.

## 1. Introduction

 The Brugada syndrome (BrS) is an inherited cardiac disorder occurring particularly in young, apparently healthy individuals and is associated with a variety of arrhythmias, mainly ventricular tachyarrhythmias that can induce syncope or sudden cardiac arrest (SCA) [1]. Patients with documented cardiac arrest should receive an implantable cardioverter-defibrillator (ICD) [2]. In the remaining subjects, the best management is still a challenge. Long-term follow-up of this subgroup of patients has revealed a low accuracy of stratification based on only one risk factor [3]. A multiparametric approach, including more than one risk factor, may increase the likelihood to predict ventricular arrhythmias that can be prevented by an ICD. 

Increased body of evidence links lethal ventricular arrhythmias with inflammatory states [4]. It is known that arrhythmic events in BrS are triggered by febrile states, independent of the etiology [5]. Amin et al. have proposed that “research is needed on the study of the *in vivo* effects of fever and its various aspects,” including “inflammatory cells and cytokines” [6]. C-reactive protein (CRP) is an acute-phase reactant, whose levels rise in response to inflammation. Serum levels of CRP have been shown to be increased soon after the occurrence of ventricular arrhythmias in other arrhythmogenic diseases like arrhythmogenic right ventricular dysplasia/cardiomyopathy (ARVD/C) [7]. Serum levels of CRP in BrS and their possible clinical implications have not been studied before. We evaluated levels of CRP in a cohort of subjects with BrS. 

## 2. Methods 

### 2.1. Study Population

Patients of our BrS registry were carefully analyzed in terms of past medical history. They were referred for three main reasons: family screening of a BrS index case, unexplained syncope, or aborted SCA. At admission, a complete clinical history, including family antecedents with emphasis on SCA, was obtained. All patients underwent physical examination and cardiac work-up that included ECG, 2D echocardiography, signal averaged ECG (SAECG), ajmaline challenge (in the absence of spontaneous type 1 ECG pattern), and at least two of the following cardiac imaging studies: right ventricular angiography, cardiac MRI, or ventricular radionuclide imaging. The diagnosis of BrS was made if ECG displayed coved-type ST-segment elevation, either spontaneously or after a sodium channel blocker challenge, in at least one right precordial lead (*V*_1_–*V*_3_) in conjunction with a documented ventricular fibrillation (VF), self-terminating polymorphic ventricular tachycardia (VT), agonal nocturnal respiration, syncope, aborted SCA, or family history (<45 years) of SCA. Asymptomatic patients were compared with symptomatic (syncope and aborted SCA). Study protocol was approved by the Institutional Review Board of Pitié-Salpêtrière Hospital, and informed consent was obtained from each participant.

### 2.2. Blood Testing

At admission, for work-up and before any cardiac intervention such as drug challenge, EP study, cardiac catheterization, or ICD implantation; as part of the routine blood sampling, CRP concentration was measured in each patient by immunoturbidimetric method (Integra 400 Roche, France). For characterization of inflammatory state, only CRP measurements were performed. Data were collected retrospectively, and we verify that no recent arrhythmic event had occurred prior to blood sample testing. In those patients with established diagnosis of BrS and carriers of an ICD, a device interrogation was performed to exclude recent (<14 days) shock discharge.

### 2.3. Study Covariates

Hypertension was defined as diastolic blood pressure ≥ 90 mmHg and/or systolic blood pressure ≥ 140 mmHg and/or a patient on antihypertensive medication. Diabetes mellitus was based on a fasting blood sugar ≥ 7 mmol/L and/or on insulin regimen or on any other antidiabetic treatment. Smoking was classified into ever and never smokers. Lipid lowering treatment was statin treatment with anti-inflammatory properties. Aspirin, even when a low dose is used, has the potential to influence CRP release [8]. Brugada-ECG pattern displayed coved-type ST-segment elevation in *V*_1_ through *V*_3_. Wide QRS was duration of QRS > 120 ms. Other covariates of interest were age, atrial fibrillation (AF), drug challenge by ajmaline test, family history of SCD, and ICD placement for high-risk status or for secondary prevention. 

### 2.4. Statistical Analysis

Continuous variables were expressed as mean ± SD if they demonstrated a normal distribution and as median (range) otherwise. Means of continuous variables that showed normal distribution were compared with the Student's *t*-test for independent samples. Continuous variables that did not show a normal distribution were compared using the Mann-Whitney *U* test. Categorical variables were summarized as proportions and compared using the *χ* ^2^  test or the Fischer exact test if cell counts were less than 5. For the multivariate model, only factors that attained statistical significance (*P* < .1) in the univariate analysis were included. A 2-tailed *P* value <.05 was considered statistically significant. Statistical analysis was performed with SPSS version 11.0.1 software (SPSS Inc).  Figure 2 was performed with Statistica v6.0 (StatSoft Inc).

## 3. Results

### 3.1. Characteristics of the Baseline Population

**Figure 1:**
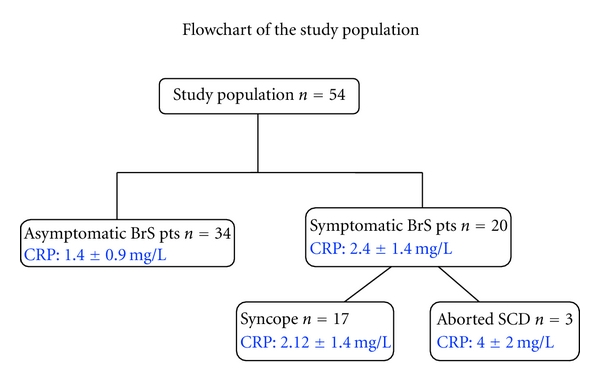
Fifty-four patients with BrS were divided into two groups according to the clinical feature. BrS: Brugada syndrome; CRP: C-reactive protein.

The study sample consisted of 54 patients, with a mean age of 45 ± 13 years old and 49 (91%) being male. Twenty (37%) were symptomatic (17 syncope and 3 aborted SCA) (Figure 1). Baseline characteristics were similar in both groups apart from the younger age of those in the asymptomatic group (40 ± 12 versus 53 ± 10, *P* = 0.001, Table 1). The prevalence of cardiovascular risk factors in the whole cohort was 15% hypertension, 15% smokers, 17% hypercholesterolemia, and 2% diabetes with no differences among groups (Table 1). Fifteen (28%) patients had family history of SCA, and 33 (61%) had spontaneous, diagnostic, coved-type ECG.

### 3.2. Comparison between Patients with and without Symptoms

Asymptomatic and symptomatic patients were comparable in gender, cardiovascular risk factors, and main ECG features (first-degree atrioventricular block, wide QRS, and coved type ST-segment elevation in right precordial leads). Mean age and CRP levels were the only parameters significantly different in univariate analysis between both groups. In Table 1 is also shown the multivariate analysis for predictors of being symptomatic. A significant value was still found for age and levels of CRP ≥ 2 mg/L (*P* = .009, CI 95% 1.56–22.08, and *P* = .018, CI 95% 1.32–19.31, resp.). 

### 3.3. Clinical Features of Patients with ICD

Implantation of an ICD started in our centre before current recommendations [2]. Therefore, 10 asymptomatic patients (29%) were implanted for high-risk status (spontaneous coved-type ST-segment elevation in conjunction with either family history of SCA or positive EP study). None of these 10 patients have experienced ICD shock, and their electrogram memories did not show ventricular arrhythmias. In 16 (80%) symptomatic patients, an ICD was implanted either before hospitalization (>3 months) or after blood sampling was taken for CRP. Four out of 16 (25%) had appropriate ICD shock during follow-up. 

### 3.4. Inflammatory Pattern and Its Relationship with Cardiac Arrhythmic Events

**Figure 2:**
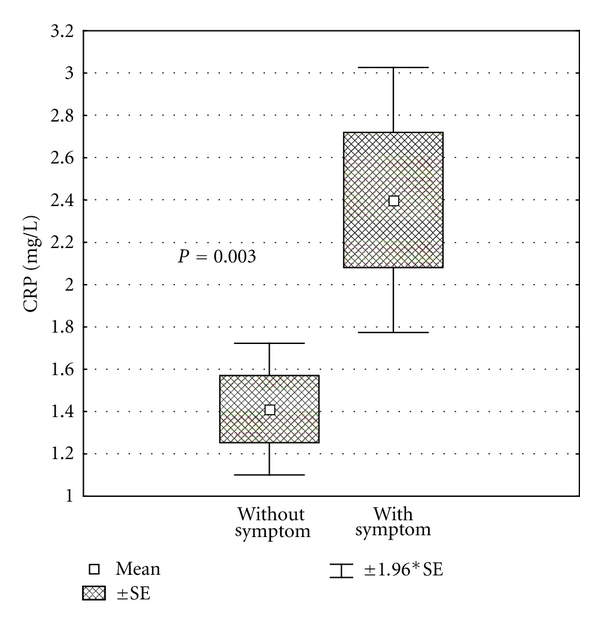
CRP concentrations in the BrS patients without (left diagram) and with (right diagram) symptoms (syncope or SCA) were significantly different. CRP: C-reactive protein; BrS: Brugada syndrome; SCA: sudden cardiac arrest.

As previously mentioned, levels of CRP were significantly different in both groups, asymptomatic versus symptomatic subjects. The mean CRP levels (Figure 2) were 1,4 ± 0,9 mg/L in asymptomatic and 2,4 ± 1,4 mg/L in symptomatic group (*P* = .003). CRP concentration ≥ 2 mg/L was an independent marker for being symptomatic (*P* = .018; 95% CI: 1.3 to 19.3) (Table 2). A CRP concentration ≥ 2 mg/L was significantly associated with type 1 ECG pattern (*P* = .022), syncope (*P* = .025), syncope and/or aborted SCA (*P* = .018), and the decision to implant an ICD (*P* = .003) in univariate analysis. After multivariate analysis, only overall symptoms (syncope and/or resuscitated SCA) remained significantly different (*P* = .039, 95% CI: 1.07–18,79) (Table 2). 

## 4. Discussion

The aim of this study was to assess the inflammatory profile measured by CRP in individuals with BrS. The study also looked at the association between past history of arrhythmic events and CRP level. 

### 4.1. Major Findings

This study shows that patients with BrS syndrome and life-threatening symptoms such as syncope or sudden death have more active inflammation than those without symptoms. Moreover, a CRP level ≥ 2 mg/L is more frequent in the presence of previous cardiac arrhythmic events. 

### 4.2. Inflammation and Cardiovascular Outcomes

CRP is a biomarker of inflammation, and high levels have been associated with an increased risk of all-cause mortality [9]. It predicts cardiovascular events such as stroke, coronary heart disease, and peripheral vascular disease [10, 11]. Serum CRP levels greater than 3 mg/L have been shown to predict these cardiovascular events [12], and anti-inflammatory agents such as statins have demonstrated a reduction of cardiovascular mortality in patients with normal lipid profile [13]. It is well known that systemic inflammation is associated with arrhythmias. This association has been extensively studied in atrial fibrillation (AF) [14]. It has been shown that increased CRP levels are associated with greater risk of AF recurrence after electrical cardioversion [15]. Moreover, dilated cardiomyopathy patients with AF have higher inflammatory activation than those without AF [16]. Ventricular arrhythmia incidence is associated with significantly elevated proinflammatory markers such as IL-6 and high-sensitive CRP in implantable cardioverter-defibrillator (ICD) patients with structural heart disease [4]. Enhanced inflammatory response is related to the development of ventricular arrhythmias after ST-elevation myocardial infarction. During acute myocardial ischemia, patients with malignant ventricular arrhythmias experience higher systemic inflammation than those without them [17]. Consistent with the later, statins which have anti-inflammatory properties [18] are associated with decreased incidence of VT [19–22]. However, recent data has suggested that inflammatory biomarkers such as IL-6, TNF-alpha, hsCRP, fibrinogen, and BNP are not predictive of intermediate-term risk of ventricular tachyarrhythmias in stable chronic heart failure [23]. Regarding the value of inflammation in predicting SCA in apparently healthy populations, controversial issues have been reported. Empana et al. have not found any association between inflammatory biomarkers and SCA in middle-aged men whereas other data suggest that CRP levels may be useful in identifying apparently healthy men who are at an increased long-term risk of SCD [24, 25]. Whether inflammatory activation is the cause or the consequence of ventricular arrhythmias is unclear. We have reported high CRP concentration in ARVD/C soon after VT with a clear tendency to decrease its level after the event [7]. 

### 4.3. Inflammation and the Brugada Syndrome

We found that a single plasma value of CRP concentration is robustly associated with either syncope or SCA in BrS. Studies assessing inflammatory patterns in BrS have reported controversial findings. One did not detect inflammatory changes in cardiac biopsies [26]. Others found locally restricted inflammation due to parvovirus B19 [27, 28]. Frustaci et al. reported histological evidence of a prevalent or localized right ventricular myocarditis in 14 out of 18 BrS patients who underwent endomyocardial biopsy [29]. The range of CRP between 1 and 3 mg/L was considered intermediate risk. In the symptomatic group of patients, the median level of CRP was 2 mg/L, which is above the value known in the general population [9–12]. Therefore, we hypothesized that this value of CRP may discriminate between individuals with and without symptoms. This hypothesis was confirmed by the finding that symptoms were more frequent in the subgroup with CRP levels ≥ 2 mg/L (Table 2). Interestingly, when adjusted for age, CRP ≥ 2 mg/L remained an independent factor for being symptomatic (Table 1). The significant association of CRP concentration with symptoms suggests that inflammation might play a role in the pathophysiology of arrhythmias in BrS. 

We consider this finding relevant for risk stratification in BrS. With an increasing number of patients being diagnosed with BrS, our knowledge has also grown in every single aspect of this disease. However, estimation of the likelihood to die suddenly remains difficult in the absence of resuscitated SCA [30]. For example, although all registries agree on the fact that patients with syncope have a poor prognosis, it has been documented that some syncope episodes in BrS patients are in fact due to a vasovagal mechanism [31, 32]. Thus, the decision to implant an ICD in syncope-related BrS patients, even in the setting of EP-induced VF, is challenging when taking into account the incidence of ICD-related complications, which is up to 28%, and death-related ICD malfunction [33, 34]. Therefore, a multiparametric approach consisting in considering diagnostic type 1 ECG pattern in conjunction with syncope and at least one other risk factor has been suggested [3]. To summarize, the risk/benefit ratio of ICD placement in this young population is difficult to estimate [35]. Therefore, there is dire need for de novo risk markers for arrhythmic events. These data show a sharp departure from the current view that BrS has a polyfactorial pathogenesis which may include inflammatory pathway. Indeed, knowledge regarding the clinical spectrum of patients with a Brugada ECG is important to implement effective risk stratification and management [36, 37]. The results presented herein may be useful to identify high-risk patients in need of primary or secondary prevention by an ICD and during the follow-up of implanted patients to detect those at risk to present ventricular arrhythmias. However, larger clinical trials are needed to confirm the increase of inflammatory activity in symptomatic Brugada syndrome.

**Study Limitations**

The strength of the study is that it is the first to our knowledge to evaluate CRP as a marker of inflammation in BrS. This may help provide a better understanding of the large spectrum of the disease. However, there are some potential limitations which merit consideration. First, we did not use the nephelometric technique assessing high sensitivity C-reactive protein (hs-CRP) which is about a 10-fold more sensitive than the immunoturbidimetric assay used in this study. Secondly, few patients in our series (11 out of 54 (20%)) have been genotyped and only one displayed a mutation in *SCN5A* gene. Therefore, a correlation between genotype and inflammation could not be established. This is a very pertinent lack of information as there is a bunch of literature showing that loss-of-function *SCN5A* mutations lead to fibrosis and structural heart disease [38, 39], emphasizing that CRP might rise via this pathway.

## 5. Conclusion 

CRP levels are increased in symptomatic patients with BrS. The causative role of inflammation in occurrence of arrhythmic events in this disease needs to be further studied. 

## Figures and Tables

**Table 1:** Baseline characteristics of asymptomatic and symptomatic BrS patients.

	Asymptomatic (*n* = 34)	Symptomatic (*n* = 20)	*P* value		95% CI
			Univariate analysis	Multivariate analysis	
Age (years)	40 ± 12	53 ± 10	.001	.009	1.56–22.08
Male	32 (94%)	17 (85%)	ns	—	
Hypertension	3 (9%)	5 (25%)	ns	—	
Hypercholesterolemia	6 (18%)	3 (15%)	ns	—	
Smokers	6 (18%)	2 (10%)	ns	—	
Diabetes	0	1 (5%)	ns	—	
Statin	1 (3%)	2 (10%)	ns	—	
Aspirin	0	1 (5%)	ns	—	
First-degree AV block	4 (12%)	5 (25%)	ns	—	
QRS > 120 ms	10 (29%)	3 (15%)	ns	—	
Type 1 ECG pattern	19 (56%)	14 (70%)	ns	—	
CRP ≥ 2 mg/L	11 (32%)	15 (75%)	.003	.018	1.32–19.31

**Table 2:** Demographic and clinical characteristics of the Brugada patients with CRP < or ≥ 2 mg/l.

	CRP < 2 mg/L (*n* = 28)	CRP ≥ 2 mg/L (*n* = 26)	*P* value		95%CI
			Univariate analysis	Multivariate analysis	
Age (years)	44 ± 14	46 ± 12	ns	—	
Male	24 (86%)	25 (96%)	ns	—	
Coved-type ECG	13 (46%)	20 (77%)	.022	ns	
VT/FV	1 (4%)	4 (15%)	ns	—	
Family history	5 (18%)	10 (38%)	ns	—	
Syncope	5 (18%)	12 (46%)	.025	ns	
Aborted SCA	0	3 (12%)	ns		
Overall symptoms (syncope and/or aborted SCA)	5 (18%)	15 (58%)	.018	0.039	1.07–18.79
ICD^†^	8 (29%)	18 (69%)	.003	ns	

CRP: C-reactive protein; VT: ventricular tachycardia; FV: ventricular fibrillation; SCD: sudden cardiac death; ICD: implantable cardioverter-defibrillator; ^†^placement of ICD was done before (>3 months) or after blood testing for serum CRP concentration.
